# Application of an Impedimetric Technique for the Detection of Lytic Infection of *Salmonella* spp. by Specific Phages

**DOI:** 10.1155/2009/259456

**Published:** 2009-11-23

**Authors:** Lara R. P. Amorim, Joana G. L. Silva, Paul A. Gibbs, Paula C. Teixeira

**Affiliations:** Escola Superior de Biotecnologia, Universidade Católica Portuguese, Rua Dr. António Bernardino de Almeida, 4200-072 Porto, Portugal

## Abstract

This study was performed to evaluate the adaption of the impedimetric method to detect the lytic infection by *Salmonella*-specific bacteriophages and to provide a higher selectivity to this rapid method in detecting *Salmonella* spp. by using specific agents. Three bacteriophages and twelve strains of *Salmonella* spp. were tested. Each of the twelve strains was used separately to inoculate TSB together with each one of the phages. The inoculum concentration was between 10^6^ and 10^7^ cfu/mL, at a cell: phage ratio of 1 : 100. From the sample analysis, based on conductance (G) measurements (37°C), the infection could be detected, by observation of both detection-time delay and distinct curve trends. The main conclusions were that kinetic detection by impedance microbiology with phage typing constitutes a method of determining whether a test microorganism is sensitive to the bacteriophage and a method to evaluate whether a lytic bacteriophage is present in a sample, by affecting bacterial growth rate/metabolic change.

## 1. Introduction

Impedance monitoring is a rapid, repeatable, and sensitive method that measures the physiochemical changes caused by bacterial growth or metabolism [1, 2]. As bacteria grow, they metabolize larger weakly charged molecules (polysaccharides, fats, proteins) and produce smaller highly charged metabolic by-products (organic acids, fatty acids, amino acids), so that by measuring the impedance parameter, one determines the bulk ionic strength variation in the growth medium. The final electrical signal is frequency dependent and also varies with temperature [3].

Microorganisms, depending on their number and metabolic activity, induce at a given moment a significant change leading to an inflection in the curve. This inflection point is referred to as a detection time (DT). The time to this detectable change will be inversely proportional to the initial number of organisms inoculated into the well [4] and also will be defined by the composition of the medium (e.g., fermentable carbohydrates will give a large change in conductivity with fermentative bacteria).

 For a food processor producing a standard product under very similar conditions each day, a calibration curve for that product can be established and used in quickly evaluating the microbial status, but only as long as the conditions of the food production, media, and temperature used in the instrument, and so forth, remain the same [5]. Using an automated impedimetric system for bacteria counts, there is even the possibility of predicting the microbial behavior in food stuffs and determining the generation times in different environmental conditions.

This and other rapid methods can be subject to interference from competitor microorganisms and/or food debris. Competitor organisms can cross-react with detection systems giving false-positive detections or can grow to a level that will “mask” target organisms [6]. The use of bacteriophages has been investigated to overcome these drawbacks to produce specific-detection regimes with minimum analysis time but maximum reliability. Bacteriophages are a type of virus that only infect and multiply in bacteria. As soon as they were known, they were suggested as therapeutic agents for combating pathogenic bacteria. Later, these viruses were applied for typing and are being investigated as an antimicrobial agent for foods [7]. From the lytic infection, release of progeny phages coincides with the destruction of the parental infected cell. It has already been described as a way of detecting bacteriophages present in lactic starter cultures by evaluating the detection-time parameter and the percentage change of conductance [8, 9].

## 2. Materials and Methods

### 2.1. Bacteriophages

Three different phages tested in this study (*ϕ* 39, *ϕ* 2/2, *ϕ* 38) were phages isolated, characterized, and held in the EC Project Phagevet-P by University of Minho, Braga, Portugal. An archival stock of each phage strain was maintained in a solution of 0.90% (w/v) NaCl under refrigerated storage. To enumerate the phage particles encountered in these experiments, the plaque assay method was performed preceded by serial dilutions in 0.90% (w/v) NaCl (Carlson, 2005).

### 2.2. Bacterial Strains

Three bacteriophage hosts were used (S1400/94, 869, 128). In addition to these *Salmonella* strains, nine more (172, 205, 002, 161, 195, 204, 152, 23, and 39) were arbitrarily selected from the ESB *Salmonella* culture collection. Working bacterial cultures were conveniently maintained at −70°C with a cryoprotectant (30% (v/v) glycerol). During the experimental period, all the isolate cultures were stored, in the laboratory of analysis, at −20°C, in TSB containing 30% (v/v) glycerol. The *Salmonella* cultures were prepared in 10 mL of tryptic soy broth (TSB, Pronadisa, Portugal), grown overnight, at 37°C.

### 2.3. Conductimetric Tests

The tests were performed in an impedance-based system (Bactometer model 64 BioMérieux Vitek, Hazelwood, Mo, USA). The archival stock of each phage strain was serially diluted in 0.90% (w/v) NaCl solution, immediately before addition to wells in the modules and insertion into the Bactometer processing unit (BPU). The concentration of bacteria giving the shortest detection time was determined in separate initial experiments. The mixture of each bacterial strain and phage suspension was tested at several cell to phage ratios. In each module (of 16 wells), one well contained sterile saline solution and another with TSB medium for sterility testing of samples; a detection time at anytime during the test period means a nonsterile assay, which also means a high probability of having contaminated samples in the same Bactometer unit. The modules were incubated at 37°C, and conductance changes in the tubes were continuously monitored at 6-minute intervals for 24 hours by the BPU. At the end of this period, continuously measured changes in conductance can be edited in report form, illustrated with “growth curves” (metabolic change curves), and stored in computer for further analysis.

### 2.4. Phage Sensitivity by “Spot Test”

Before performing the Bactometer tests, the susceptibility of the 12 *Salmonella* strains to infection by the tested phages had been recorded using the “spot test” method. This method is based in the plaque assay described by d'Herelle for phage enumeration [10]. Instead of mixing the phages with the bacterial cells to be poured in a layer of soft agar, aliquots of a phage suspension are placed on the film of bacteria growing in “top agar” surface. When the infection is detected by forming a halo, that means that the isolate is susceptible to the phage and produces plaques (positive indication +), while a negative (−) indicates that no plaques were observed (Table 1). It was expected to obtain similar results using the “spot test” or the conductimetric test.

## 3. Results

### 3.1. Optimization of the Conductance Method

The inoculation levels of bacterial cells and cell to phage ratio are operational parameters, which were evaluated prior to the large scale test of effects of bacteriophage on *Salmonella *spp. In the absence of phage, all *Salmonella* strains displayed similar patterns of conductivity change during the course of incubation. Further, for almost all strains, detection times were inversely proportional to the inoculation levels. The shortest detection times were obtained at inoculation concentrations of 10^6^ and 10^7^ cfu/mL. To obtain the detection time values for analysis, this experiment was done in triplicate for the bacteriophage host strains (S1400/94, 869', and 128' isolates; see Table 2).

**Figure 1:**
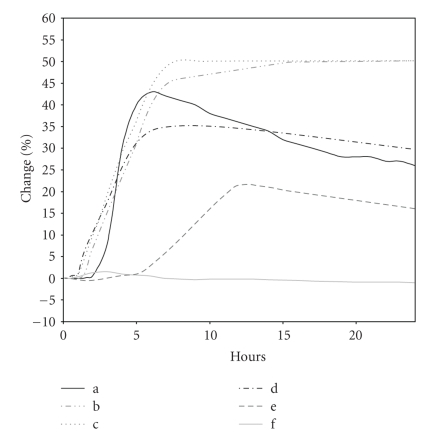
Detectable change in electrical characteristics checked at several bacteria densities. There were considered the mean values of duplicates from three experiments made for a representative phage (2/2) and its bacterial host (S1400/94): (a) 10^4^ cfu/mL; (b) 10^5^ cfu/mL; (c) 10^6^ cfu/mL; (d) 10^7^ cfu/mL; (e) 10^8^ cfu/mL; (f) 10^9^ cfu/mL.

The largest electrical changes were observed for inocula of 10^5^ and 10^6^ cfu/mL, registering 50% of change in the signal since initiation (Figure 1). It must also be considered that at inoculation concentrations between 10^4^ and 10^7^ cfu/mL, the curve patterns look like the characteristic “bacterial growth curve” shape expected from Bactometer graphs. The same cannot be observed for the higher concentrations, and there was a delay in detection times.

In the phage infection experiments, 10^6^ cfu/mL inoculation concentration was used. Throughout this study of infection detection using the Bactometer, in the absence of phage, all *Salmonella* strains displayed similar patterns of conductivity change during growth, at this chosen inoculation concentration.

Another optimization of this method was performed by studying the effect of cell to phage ratio on the conductance curves of each host strain of *Salmonella, *using the corresponding phage. Starting with the 10^6^ cfu/mL inoculation concentration as the base level, different concentrations of phage suspension were added. Each host strain was tested against its specific phage so that the comparison between data resulting from samples containing no phage and samples inoculated with a known concentration of phage allowed the detection of lytic infection.

**Figure 2:**
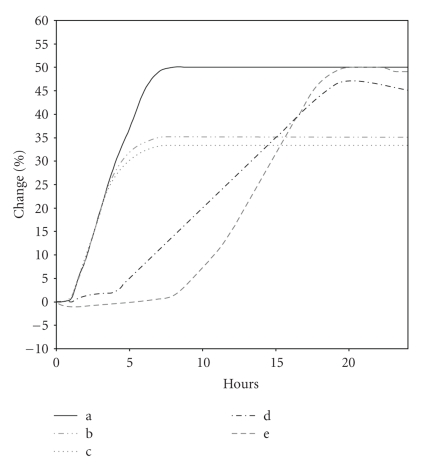
Effect of cell : phage ratio on the detection times and percentage change of conductance of 2/2 bacteriophage host S1400/94. These values were obtained from three experiments, in duplicate. Cell : phage ratio—(a) 1 : 0; (b) 1 : 0.1; (c) 1 : 1; (d) 1 : 10; (e) 1 : 100.

Conductance changes after the detection time were the only parameters related to phage concentrations. The percentage change values of conductance at about 5 hours after inoculation were inversely related to the initial numbers of plaque-forming units per mililiter (pfu/mL, Figure 2). From the cell to phage ratio study, it was observed that detection times were affected by phage present in cultures but did change proportionally to initial phage numbers. The samples infected with (b) 1 : 0.1 ratio and (c) 1 : 1 pfu/mL, behaved as the control (a) (no phage control) within 4 to 5 hours after inoculation, but conductance changes stopped after 6 to 8 hours. 

As there was a significant prolongation of the detection time at a cell : phage ratio of 1 : 100 compared with the control and the other samples, indicating a highly inhibitory action on bacterial growth, this was the chosen cell : phage ratio for further experiments. Until the third hour after inoculation, it was not possible to distinguish between the assays; different concentrations under the same exponent show this type of variation patterns. Besides being a very stable assay with very low standard deviation, like the others, its pattern allows the establishment of a numeric relation between not only the detection time and the control assay, but also by integral calculation (calculation of area). It is also important to mention that minor detection times are desirable for a rapid assay. Similar results were obtained for the 869' and 128' host strains with their specific phages, 39 and 38, respectively, as is shown in Table 3.

### 3.2. Definition of Test Sensitivity and Specificity

The conductance technique allowed detection of the phage in all tested samples by causing a delay in detection time and affecting the percentage change of conductance curves, mainly during the first few hours, shown in Table 4. As a result, the conductance technique can be characterized as a sensitive method. 

When a bacterial strain and its lytic phage are inoculated at the same time in a sample and cells are infected, it was expected to observe a large delay in detection time and smaller total conductance changes, and consequently, a larger differential between the area between the control curve and that of the sample than when dealing with a noninfective phage; in this case, these values should be small. 

Expecting similar results from conductance test results and the ability to form plaques on soft agar lawns, a comparison between the results of the two tests was established, as shown in both Tables 1  and 3. The sensitivity of the conductance method is defined as the percentage of samples that were found to show differences in conductance curves between control and phage-infected cultures. The specificity is defined as the percentage of samples that were found to correspond to plaque-forming test results. However, the specificity cannot be computed since both lytic and nonlytic phages produced a delay in DT; the Bactometer method appeared to show a positive result (delay in DT and reduction in total conductance changes), for all phage-*Salmonella *combinations. Among the twelve strains, only five (S1400/94', 869', 128', 172, and 152) showed the expected results in the Bactometer test with their respective lytic phages. However, the remaining seven strains also showed differences in their conductance curves with the nonplaque-forming phages. As an example, the phage 39 infected its host, 869' in the plaque assay, but did not produce plaques on the 161 isolate. Observing the Bactometer results, the curves registered do not characterize these two strains in the same way; that is, they show similar curve patterns, based on both detection time and differential values for phage 39.

Within the Bactometer positives, no relationship was found between the plaque size and the detection time, nor with the differential between curves. For instance, the phage 38 had a similar infective behaviour in plaques on that of S1400/94 and 172 isolates, although, with regard to conductance measurements, this phage seems to affect much more that of 172 isolate's metabolic behaviour than the S1400/94'.

## 4. Discussion

From analysis of the data, it may be concluded that this method is not specific for detecting lytic phages, although it is sensitive, rapid and may detect infection and alteration of host cell metabolism but not produce cell lysis and production of mature phage particles. Nevertheless, it is a method that, incorporating conductance measurement and specific bacteriophages for the identification of *Salmonella* spp. and the detection of a delay in detection time, was simple and capable of automation. Kinetic detection by conductance microbiology with a pathogen-specific phage constitutes a method of determining whether an unknown test microorganism is the target organism and also a technique to determine whether a specific bacteriophage is present in a sample, by affecting bacterial growth rate/ metabolic change.

Within this work, it was not possible to conclude, for the purpose of determining whether a test isolate is susceptible to a *Salmonella*-specific bacteriophage, that the conductance based assay is preferable to a plaque assay. A hypothesis for the difference observed between plaque formation and a Bactometer result could be that, although a phage may be capable of infecting a strain of *Salmonella*, for some reason the infected cell does not produce complete phage particles and is not lysed at the end of the phage cycle. However, the phage genome may still have the ability to redirect the metabolic processes of the infected cell thus leading to a delay in detection time. It is possible also that such an infection could lysogenise the bacterial cell.

It was not possible to compare these two different methodologies (spot test in plaques and Bactometer monitoring) to evaluate the lytic infection by phages in media with distinct physical structures; differences between phage growth in plaques versus broth occur throughout the enlargement phase of plaques during which the physical structure of solid media slows both phage and host diffusion, prevents gross environmental mixing, and probably gives rise to local phage multiplicities that are much higher than one observes during the majority of phage production in broth. So, maybe it is wrong to classify the Bactometer method as less specific comparing it with the spot (plaque-forming) test. To be sure of this, new approaches must be used to evaluate the performance of bacteriophages tested in this work, [11], have tried high-performance liquid chromatography, and [12] developed the immunomagnetic separation-bacteriophage assay, for instance. 

The conductance parameter was the one chosen from the three electrical changes that can be measured in the Bactometer, although other authors have used other signals [8, 9, 13–15]. This method also requires further studies related to the characteristics of various strains and phages, and also verification of the expected detection of lower phage concentrations, as described by [8].

In further studies, using the Bactometer or exploring a different kind of method, a set of determinations can be made, such as the initial concentration of bacteriophages and some kinetic factors of the lytic infection (MOI, phage-coded functions and increase of the number of bacterial isolates). Indeed, the nutritional status of the bacterium, if the phage utilizes the bacterial machinery maximally for its reproduction, can significantly affect the burst size, but the phage-coded functions such as polymerases and regulatory proteins essential for phage production (their inherently low efficiency may not permit the phage to make full use of bacterial resources and consequently the burst size may be limited) plays a greater influence on the burst size [16].

Future studies should investigate whether bacteria are infected and produce a delay in the Bactometer detection time by nonlytic phages (i.e., not producing plaques in the spot test), why does this occur? Possible avenues of research could be: (a) attachment of phage and injection of DNA but for some reason the phage genome does not code for production of new phages, (b) possible degradation of phage DNA by restriction enzymes, or (c) sufficient modification of bacterial metabolism to result in a delay in DT, but not production of mature phages. Each of these (and other) hypotheses should be studied to understand better our results in the Bactometer.

## Figures and Tables

**Table 1:** Susceptibility of tested bacteria to phages according to spot test method.

Isolate	*ϕ* 2/2	*ϕ* 39	*ϕ* 38
S1400/94'	+ (6.33)*	+ (7.21)	+ (7.08)
869'	+ (11.8)	+ (13.5)	+ (12.1)
128'	+ (12.9)	+ (12.6)	+ (12.4)
172	+ (10.8)	+ (8.50)	+ (8.00)
205	+ (6.83)	−	−
002	−	−	−
161	+ (7.33)	−	−
195	+ (9.45)	−	−
036	−	−	−
152	+ (12.5)	+ (10.0)	+ (9.10)
039	+ (3.38)	−	−
023	+ (3.42)	−	−

*The values presented between parentheses correspond to average regarding the plaque size.

A positive indication (+) means that the isolate is susceptible to the phage and produces plaques, while a negative (−) indicates that no plaques were observed.

**Table 2:** Effect of cell concentration on the detection times.

	Detection time (hours)		
Cell concentration	S1400/94	869'	128'
10^4^ (a)	2.5 ± 0.1	2.6 ± 0.2	2.5 ± 0.2
10^5^ (b)	1.8 ± 0.2	2.0 ± 0.1	1.9 ± 0.2
10^6^ (c)	1.3 ± 0.1	1.1 ± 0.2	1.3 ± 0.2
10^7^ (d)	0.8 ± 0.1	0.8 ± 0.2	0.9 ± 0.1
10^8^ (e)	3.8 ± 0.1	3.5 ± 0.2	3.7 ± 0.2
10^9^ (f)	—	—	—

Note: Means and standard deviations are based on three experiments in duplicate.

**Table 3:** Effect of cell to phage ratio on detection times.

	Detection time (hours)		
Cell : phage ratio	S1400/94	869'	128'
1 : 0 (a)	1.0 ± 0.1	1.1 ± 0.2	1.0 ± 0.2
1 : 0.1 (b)	1.1 ± 0.2	1.0 ± 0.3	0.9 ± 0.3
1 : 1 (c)	1.0 ± 0.3	1.1 ± 0.3	0.9 ± 0.3
1 : 10 (d)	4.8 ± 0.2	4.0 ± 0.2	4.9 ± 0.2
1 : 100 (e)	8.0 ± 0.3	8.1 ± 0.3	8.3 ± 0.3

Note: Means and standard deviations are based on three experiments in duplicate.

**Table 4:** Results obtained from conductimetric tests.

	Detection time (hours)				Differential between curves (area) (mm^2^)*		
Bacteria	No phage	*ϕ* 2/2	*ϕ* 39	*ϕ* 38	*ϕ* 2/2	*ϕ* 39	*ϕ* 38
S1400/94	1.3 ± 0.2	4.5 ± 0.1	4.0 ± 0.2	2.5 ± 0.5	5.6 ± 1.3	2.7 ± 0.2	3.0 ± 0.2
869'	1.8 ± 0.2	6.5 ± 0.4	2.1 ± 0.2	3.1 ± 0.5	7.7 ± 0.4	4.3 ± 0.5	5.7 ± 0.4
128'	1.5 ± 0.1	6.6 ± 0.7	4.0 ± 0.5	2.6 ± 0.4	6.9 ± 0.5	6.2 ± 0.4	4.5 ± 0.3
172	1.1 ± 0.4	6.6 ± 0.9	7.4 ± 0.3	8.5 ± 0.5	9.0 ± 0.9	7.6 ± 1.6	8.4 ± 0.1
205	1.5 ± 0.1	7.0 ± 0.2	6.7 ± 0.3	6.8 ± 0.1	8.3 ± 0.8	4.2 ± 0.6	6.7 ± 0.3
002	1.3 ± 0.2	7.2 ± 0.7	7.2 ± 0.2	5.0 ± 0.2	8.7 ± 0.7	4.4 ± 0.3	4.7 ± 0.2
161	1.8 ± 0.2	8.0 ± 0.4	3.1 ± 0.3	2.5 ± 0.3	7.8 ± 0.6	4.7 ± 0.3	3.4 ± 0.5
195	1.7 ± 0.4	6.9 ± 0.3	6.1 ± 0.3	7.5 ± 0.2	6.4 ± 0.6	6.5 ± 0.1	13.4 ± 0.3
036	1.4 ± 0.2	7.5 ± 0.3	6.1 ± 0.5	7.2 ± 0.3	9.2 ± 0.6	4.1 ± 0.2	8.4 ± 0.2
152	1.3 ± 0.3	6.8 ± 0.7	6.7 ± 0.4	7.0 ± 0.4	8.6 ± 0.7	8.1 ± 0.5	6.6 ± 0.6
039	2.0 ± 0.3	6.5 ± 0.8	6.5 ± 0.2	6.5 ± 0.6	8.8 ± 0.5	7.1 ± 1.1	8.4 ± 0.2
023	1.4 ± 0.4	4.0 ± 0.6	4.5 ± 0.2	4.1 ± 0.3	4.1 ± 0.3	3.1 ± 0.6	5.9 ± 0.3

Note: Means and standard deviations are based on three experiments in duplicate.

*The differential between curves is obtained by calculating the area between the control curve (sample with no phage) and that of the sample (with a phage).
